# A Rare Collection

**Published:** 1872-11

**Authors:** 


A RARE COLLECTICN.—Dr. Jackson exhibited to the Suffolk District Medical Society a photograph of a collection of three hundred needles, which had been removed from the body of an insane woman, at the asylum in Utica, New York. There was no history as to the introduction of the needles before she entered the asylum, nor was she detected in their introduction after entering the hospital. A few weeks after her admission, however, an inflammation of the breast was developed, and at the place of the pointing of the pus a needle was discharged—the first of the three hundred which were removed from various portions of the body.

Dr. Jackson also showed to the Society another collection, with a history similar to the above, which were removed from the body of a negro woman, of Nantucket, many years ago. *Boston Medical and Surgical Journal.*

HYDRATE OF CHLORAL IN THE PREVENTION OF SEA SICKNESS.—In a letter to the editor of the *London Lancet*, November, 1872, Dr. J. C. Ogilvie Will, of Aberdeen, corroborates the testimony of other medical gentlemen as to the use of hydrate of chloral in the prevention of sea sickness. The drug failed in only one case out of upwards of eighteen during a voyage from Aberdeen to New York, whilst other passengers who did not take the chloral, generally suffered as is usual. He also, at another time afterwards, prescribed the remedy for a young lady, in whom the symptoms were promptly arrested and did not reappear during a passage of two days. Light and easily digested food is recommended before sailing, and the patient is ordered to lie down as soon as the symptoms are felt, and take thirty grains of the drug dissolved in sweetened water; the dose to be repeated in twenty minutes, if relief is not obtained.

